# Label-Enhanced Surface Plasmon Resonance: A New Concept for Improved Performance in Optical Biosensor Analysis

**DOI:** 10.3390/s131115348

**Published:** 2013-11-08

**Authors:** Niko Granqvist, Anders Hanning, Lars Eng, Jussi Tuppurainen, Tapani Viitala

**Affiliations:** 1 Division of Biopharmaceutics and Pharmacokinetics, Faculty of Pharmacy, University of Helsinki, P.O Box 56, Helsingin Yliopisto 00014, Finland; E-Mail: tapani.viitala@helsinki.fi; 2 BioNavis Oy, Elopellontie 3C, Ylöjärvi 33470, Finland; E-Mail: jussi.tuppurainen@bionavis.com; 3 Episentec AB, Solna Strandväg 3, Solna 171 54, Sweden; E-Mails: anders.hanning@episentec.com (A.H.); lars.eng@episentec.com (L.E.)

**Keywords:** biosensor, surface plasmon resonance, SPR, label, sensitivity, specificity

## Abstract

Surface plasmon resonance (SPR) is a well-established optical biosensor technology with many proven applications in the study of molecular interactions as well as in surface and material science. SPR is usually applied in the label-free mode which may be advantageous in cases where the presence of a label may potentially interfere with the studied interactions *per se*. However, the fundamental challenges of label-free SPR in terms of limited sensitivity and specificity are well known. Here we present a new concept called label-enhanced SPR, which is based on utilizing strongly absorbing dye molecules in combination with the evaluation of the full shape of the SPR curve, whereby the sensitivity as well as the specificity of SPR is significantly improved. The performance of the new label-enhanced SPR method was demonstrated by two simple model assays: a small molecule assay and a DNA hybridization assay. The small molecule assay was used to demonstrate the sensitivity enhancement of the method, and how competitive assays can be used for relative affinity determination. The DNA assay was used to demonstrate the selectivity of the assay, and the capabilities in eliminating noise from bulk liquid composition variations.

## 1. Introduction

Label-free biosensors based on a variety of physical transduction principles, e.g., optical, electrochemical and gravimetric transduction, are well-established research tools. Among optical biosensors, the sensors based on surface plasmon resonance (SPR) have gained the most widespread acceptance within application areas such as protein-protein interaction studies and drug screening, including fragment screening [1–3]. Ever since the pioneering SPR biosensor work of Liedberg *et al.* [4] and of Flanagan and Pantell [5], considerable scientific effort has been invested into improving the analytical performance–in particular the analytical sensitivity–of SPR instruments. Today, SPR is a highly mature technology, and there are thousands of research papers published on applications of SPR-based biomolecular interaction analysis [6,7].

The most distinct advantage of label-free sensor methods is the actual absence of a label that may potentially alter the chemical properties of the analyte and interfere with the biochemical binding event. However, it is well known that this absence of a label inherently causes SPR, like other label-free methods in general, to display certain performance limitations, most notably concerning sensitivity and specificity. Even though the sensitivity of SPR instruments has been steadily refined over the last 20 years, it still does not compare favourably with label-based methods like e.g., fluorescence and radiochemical methods. The sensitivity of SPR instruments may often be inadequate when dealing with small molecules, low concentrations, weak or slow binding events, or in cases where there are limited amounts of biochemically active binding partners immobilized on the sensor surface [8–10]. It has been discussed [11] if the sensitivity of SPR instruments can be further improved or whether the theoretically achievable sensitivity limit has already been reached. Additionally, in many practical situations, noise sources such as temperature and pressure variations and variations in the composition of the bulk liquid, may dominate over pure instrument noise and thus limit the practically achievable detection limit [12]. When it comes to specificity, SPR, like most other label-free methods, is a universal detection method that detects any substance that binds to the surface, irrespective of the identity of the substance. Non-specific binding of unidentified substances, notably proteins, from the sample solution that interferes with the detection of the actual analyte is a very common problem [13].

Label-based methods, e.g., fluorescence, generally show superior sensitivity and specificity as compared to label-free methods. However, fluorescence may show other disadvantages apart from the potentially interfering presence of the label *per se*. Fluorescence-based sensor methods generally show inferior quantitative robustness, and are hampered by phenomena like quenching, photobleaching, and environmentally induced variations of the quantum yield [14]. In particular, in the case of SPR-excited fluorescence, the presence of the gold surface of the SPR sensor slide, which is a prerequisite for the strong, exciting evanescent field of the sensor, may cause severe quenching of the fluorophore [3,15]. Also, the highly specific nature of fluorescence detection may in many cases prove to be a limitation, since the binding of non-fluorescent substances, e.g., in the preceding immobilization step, cannot be monitored or quantified.

**Figure 1.**
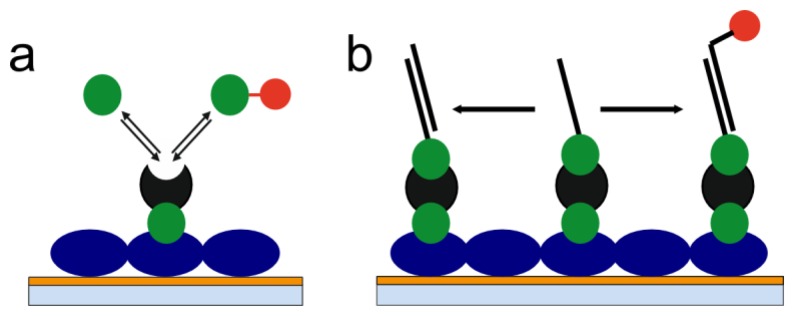
Artistic illustrations of the assays used in the study. (**a**) Small molecule assay: Sensor consisting of BSA (blue) and avidin (black), used in the competitive assay of biotin (green) and labelled (red) biotin; (**b**) DNA hybridization assay: The sensor had the same basic structure as in the small molecule assay, but single-stranded DNA was bound through biotin to the surface-bound avidin, and either non-labelled or labelled complementary DNA was introduced as the sample.

With a view on the limitations of label-free as well as label-based methods presented above, we have developed the concept of label-enhanced SPR sensing. The concept is based on utilizing strongly absorbing dye molecules in combination with the evaluation of the full shape of the SPR curve. The sensitivity, on a mass basis, is significantly enhanced as compared to conventional label-free SPR. The influence of noise factors like temperature, pressure, and bulk liquid composition variations is also significantly reduced by using label-enhanced SPR sensing. The specificity, with respect to the label, is very high, which reduces the problem of non-specific binding. Additionally, label-enhanced SPR can be run simultaneously and in parallel with label-free SPR on standard SPR hardware. This means that all capabilities of conventional SPR, like universal monitoring of all binding steps, including immobilization, and the full real-time and kinetics capabilities, are completely retained. Consequently, all the advantages of label-free and label-based sensing are combined on one single instrument platform. In this paper, we outline the theory and the principles of label-enhanced SPR, and demonstrate the improved sensitivity and specificity using two simple biochemical model systems: a small molecule assay and a DNA hybridization assay (Figure 1).

## 2. Experimental Section

### 2.1. Theory

The full quantitative theory of SPR, which takes its origins in Maxwell's equations, has been dealt with in considerable detail elsewhere [16–19], therefore, only a simplified approach will be used here to outline the foundation of label-enhanced SPR. The approach is based on the Kretschmann optical configuration (prism coupling), which is the predominant optical configuration of SPR instruments today. The optical system consists of a glass sensor slide or prism, which is covered by a thin layer of gold in immediate contact with a dielectric medium, *i.e.*, the sample, into which the evanescent field of the surface plasmon wave extends. Light from a monochromatic light source is guided through the prism on the back of the gold layer and the intensity of the reflected light from the gold layer is monitored in the angular domain. This results in a graph of intensity as a function of angle, which shows a pronounced minimum due to light absorption and excitation of surface plasmons at a specific combination of light wavelength and reflection angle. This function is called the SPR curve or the SPR dip. In this case, the following equation is valid:

(1) $${\text{n}}_{\text{p}}\;\text{sin}\;\theta =\text{Re}[{\left(\varepsilon_{\text{m}}\varepsilon_{\text{s}}/(\varepsilon_{\text{m}}+\varepsilon_{\text{s}})\right)}^{½}]$$

where n denotes refractive index, θ denotes the angle of minimum intensity (due to surface plasmon excitation), ε denotes the complex permittivity, and subscripts p, m, and s denote the prism, metal, and sample, respectively. Equation (1) is the quantitative equation used in conventional, label-free SPR. n_p_ and ε_m_ are constant at a constant wavelength. The imaginary part of ε_s_ can be neglected for non-absorbing samples, which reduces ε_s_ to n_s_^2^. Consequently, the angle θ becomes a simple function of n_s_, *i.e.*, the weighted average of the refractive index in the evanescent field zone, which is a measure of the amount of substance bound to the sensor surface. For absorbing samples, θ depends also on the imaginary part of ε_s_, *i.e.*, on the absorption coefficient a_s_ of the sample. However, it is found both from theory and from experiments that this dependency is rather small [20–22]. Thus:

(2) $$\theta =\text{f}\left({\text{n}}_{\text{s}},{\text{a}}_{\text{s}}\right)\approx \theta_{0}+{\text{k}}_{1}\left({\text{n}}_{\text{s}}–{\text{n}}_{\text{s},0}\right)+{\text{k}}_{2}\left({\text{a}}_{\text{s}}–{\text{a}}_{\text{s},0}\right)$$

where the second expression is a linear approximation in the shape of a two-dimensional Taylor expansion around an initial value θ_0_ and k_1_ and k_2_ are simple constants. The linear approximation is usually assumed to be valid within the normal working range of SPR [23]. However, even though the influence of the absorbance term in Equation (2) is rather small, an absorbing sample will influence not only the angle θ, but the entire shape of the SPR curve. The main effect of light absorption in the sample is a broadening of the curve due to attenuation of the surface plasmons; the higher the absorption, the higher the attenuation and the curve broadening. The full derivation of the shape of the curve is quite lengthy [19], and it is sufficient to state here that the dip width depends primarily on the absorbance a_s_ and to a smaller extent on the refractive index n_s_ according to the approximate equation:

(3) $$\text{W}=4\gamma /({\text{n}}_{\text{p}}\;\text{cos}\;\theta )$$

where W is the angular half-width of the SPR curve (the full angular width of the SPR curve at 50% reflectivity) and γ is a factor that depends primarily on the absorbance of the sample. In analogy with Equation (2), this can be written as:

(4) $$\text{W}=\text{g}\left({\text{n}}_{\text{s}},{\text{a}}_{\text{s}}\right)\approx {\text{W}}_{0}+{\text{k}}_{3}\left({\text{n}}_{\text{s}}–{\text{n}}_{\text{s},0}\right)+{\text{k}}_{4}\left({\text{a}}_{\text{s}}–{\text{a}}_{\text{s},0}\right)$$

Note that the n_s_ term dominates in Equation (2), while the a_s_ term dominates in Equation (4). Equations (2) and (4) may now be differentiated:

(5) $$\begin{array}{l} \Delta \theta ={\text{k}}_{1}\;\Delta {\text{n}}_{\text{s}}+{\text{k}}_{2}\;\Delta {\text{a}}_{\text{s}}\\ \Delta \text{W}={\text{k}}_{3}\;\Delta {\text{n}}_{\text{s}}+{\text{k}}_{4}\;\Delta {\text{a}}_{\text{s}} \end{array}$$

Equation (5) is a simple linear equation system with two unknowns: n_s_ and a_s_. Hence, both unknowns can be solved by measuring Δθ and ΔW. The constants k_1_–k_4_ are empirical constants that appear through the Taylor expansions. They depend on the measurement wavelength, the optical properties of the prism, and the initial value of the sample refractive index, but are true constants at a defined experimental setup.

The n_s_ term is the ordinary refractive index signal measured in conventional SPR. However, a significant improvement of the sensitivity can be obtained by using label-enhanced SPR and by selecting the label to be a dye molecule with an anomalously high refractive index at the measurement wavelength [24–26]. According to the Kramers-Kronig relations of fundamental optics [25], the maximum value of the refractive index of an absorbing compound appears at a slightly longer wavelength than the absorption maximum.

The a_s_ term is a measure of the absorbance, *i.e.*, a highly specific measure of the amount of dye-label adsorbed onto the sensor surface. This is the basis of the high specificity of label-enhanced SPR. Except in the very unlikely case of non-specific binding of coloured substances from the sample solution, the a_s_ term is unaffected by non-specific binding. Also, since most sources of noise, e.g., temperature, pressure, and bulk composition variations, do not contribute to the a_s_ term, this term will show very low system noise, which also contributes to the improved analytical sensitivity.

Thus, in order to maximize the sensitivity of label-enhanced SPR, it is advantageous to select the dye label to have as high a refractive index and as high an absorption coefficient as possible at the measurement wavelength. In order to fulfil both these criteria, the dye should be selected to have an absorption maximum at a slightly shorter wavelength than the SPR measurement wavelength at hand. However, from a more practical point of view, other properties of the label also come into play, like e.g., solubility, molecular weight, molecular charge, chemical reactivity, hydrophobicity/hydrophilicity, chemical stability, photostability, purity, and cost of synthesis.

### 2.2. Materials

Sodium hydroxide, 20× saline sodium citrate buffer (SSC), sodium dodecyl sulphate (SDS), dimethylsulfoxide (DMSO), sucrose, biotin (native biotin), avidin, and biotin-labelled bovine serum albumin (biotin-BSA) were obtained from Sigma-Aldrich (Helsinki, Finland). Biotin-labelled 25-mer DNA oligonucleotide probe (probe DNA) and complementary 25-mer DNA oligonucleotide (native DNA) were obtained from IDT (Helsinki, Finland). Biotin labelled with dye B12 (labelled biotin) and complementary 25-mer DNA oligonucleotide labelled with dye B10 (labelled DNA) were obtained from Episentec (Solna, Sweden). 2×SSC used in the experiments was diluted from 20× to a 2× working solution (SSC, 20 mM sodium citrate, 300 mM NaCl, pH 7.4) using Milli-Q grade water with a resistivity of 18.2 MΩ·cm.

SPR gold sensor slides were obtained from BioNavis (Tampere, Finland). The SPR sensors were used immediately after cleaning with a hydrogen peroxide-ammonia-water solution according to the protocol suggested by the manufacturer. Shortly, the sensors were cleaned in a boiling 1:1:5 solution of hydrogen peroxide-ammonia-water for 10 min, washed carefully with plenty of water, and wiped with a cotton-tipped applicator wetted with a 4% SDS solution followed by drying with nitrogen. The SPR instrument used in the measurements was a BioNavis SPR Navi 200-L, equipped with 785 nm and 670 nm light sources. The liquid handling in the experiments was performed using the built-in peristaltic pump and 12-port chromatography injector, and the flow cell used had 1 μL inner volume. All measurements were performed at 25 °C.

The SPR data was analysed using the SPR Navi Data Viewer and the EpiGrammer™ (Episentec) programs. Conventional or “standard” SPR sensorgrams, monitoring purely the refractive index n_s_, are reported as Δθ values in units of degrees. Enhanced sensorgrams, displaying the absorption coefficient after solving the linear equation system of Equation (5), are reported as a_s_ values. The a_s_ values may mathematically also be reported in units of degrees. However, after solving Equation (5), in which the constants may vary somewhat due to varying experimental conditions, it is not physically meaningful to compare these a_s_ units to “standard” degrees. Therefore, a_s_ values are reported in arbitrary units (a.u.) instead.

### 2.3. Small Molecule Assay

The sensor slides for the small molecule assay were prepared *in situ* in the instrument by using a freshly cleaned sensor slide. 2×SSC with 5% DMSO added for solubility enhancement was used as the running and sample buffer for all measurements. The buffer flow was 50 μL/min, and the duration of all sample injections were 2 min. The sensor surfaces were first functionalized with biotin-BSA by injecting 100 μg/mL of biotin-BSA and allowing the protein to spontaneously self-assemble on the gold. This was followed by two subsequent injections of 100 μg/mL of avidin. After the baseline had stabilized, either pure samples of native biotin or labelled biotin, or samples of mixed native biotin and labelled biotin (total biotin concentration 50 μM; mixing ratios 0.0, 0.11, 0.25, 0.4, 0.6 and 1.0 native/labelled), were injected. The concentrations used were high compared to the biotin affinity, and were meant to fully saturate the binding during the relatively short injection time. Since the sensor slides were not regenerable after the biotin injections, a new sensor slide was used for each mixing ratio, and since the amount of avidin varied between sensor slides, the signals of the analyte injections were normalized relative to the avidin binding signal for each sensor slide (Table 1). The use of non-regenerable sensor slides is of course not very practical in a high-throughput assay, but served well to demonstrate the performance in the present experiments. The biotin binding signal was averaged over a two minute interval after the passing of the sample injection pulse through the system.

### 2.4. DNA Hybridization Assay

The sensor surface for the DNA detection assay was prepared *in situ* in the instrument by using a freshly cleaned sensor slide. 2×SSC was used as the running and sample buffer for all measurements. The buffer flow used was 100 μL/min, and the duration of all sample injections was 2 min. The sensor surface was first functionalized with biotin-BSA by injecting 100 μg/mL of biotin-BSA and allowing the protein to spontaneously self-assemble on the gold. This was followed by two subsequent injections of 100 μg/mL of avidin and 25 μg/mL of biotinylated probe DNA. After the baseline had stabilized, several subsequent injections of native DNA and labelled DNA at a concentration of 25 μM were performed, with 10 mM sodium hydroxide regeneration injections between the sample injections.

In the noise reduction experiments (Section 4.4), hybridization of labelled DNA oligonucleotides was performed as above, but with sucrose in the concentration range 0%–0.3% added to the DNA sample solutions.

## 3. Results and Discussion

### 3.1. Small Molecule Assay: Sensitivity

**Figure 2.**
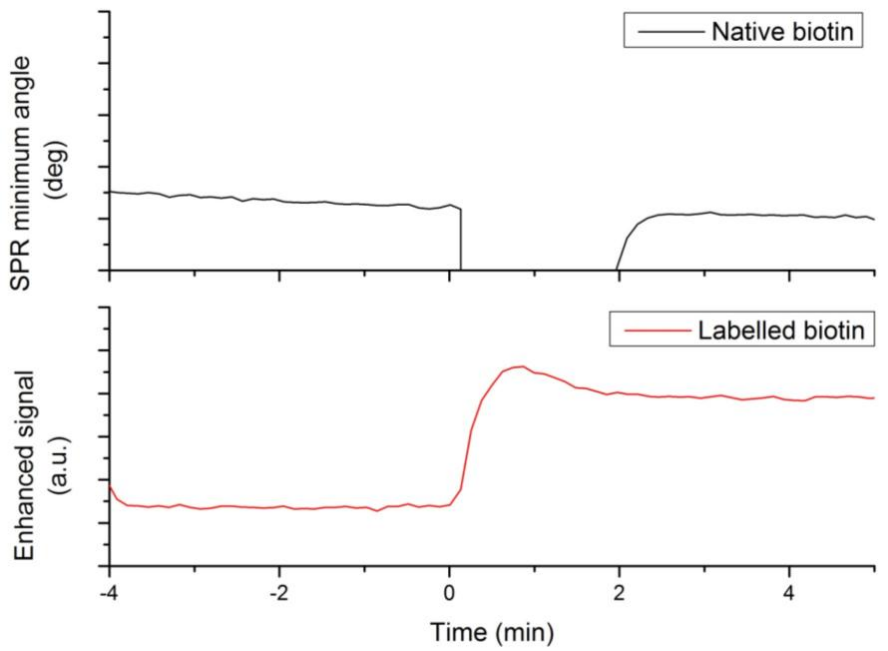
Comparison between signal generation of native biotin (upper panel) and labelled biotin (lower panel), demonstrating the sensitivity enhancement of the label-enhanced SPR method. The standard sensorgram of native biotin (400 μM in PBS) does not produce any detectable signal after the injection (the negative peak is due to the bulk effect), while the enhanced sensorgram of labelled biotin (25 μM in running buffer) produces a strong signal with a signal-to-noise ratio of about 100.

A first set of experiments was designed to demonstrate the enhanced sensitivity of label-enhanced SPR. Firstly, a 400 μM sample of native biotin was injected onto an avidin sensor slide, and the sensorgram was registered in conventional SPR mode. The result is shown in the upper panel of Figure 1. After the initial baseline, there is a large, negative injection peak, but after the sample plug has passed, there is no detectable signal from biotin binding. Secondly, a 5 μM sample of labelled biotin was injected onto another avidin sensor slide, and the sensorgram was registered in the enhanced, absorbance-measuring mode. The result is shown in the lower panel of Figure 2. There is a small overshoot from the injection, but after the sample plug has passed, there is a strong, stable signal from the binding of labelled biotin. The signal-to-noise ratio of the binding signal, as compared to the noise (standard deviation) of the initial baseline, is about 100. Thus, in effect, the analytical sensitivity with respect to biotin binding is enhanced 100×.

It is to be noted that the avidin sensor slide provides a low-capacity surface, consisting essentially of a monolayer of avidin molecules, and consequently a low signal is to be expected upon biotin binding. Molecules of the size of biotin (244 Da) can, under optimized conditions, be detected using conventional SPR when binding to a high-capacity surface based on a three-dimensional, hydrophilic network (e.g., a dextran matrix) [27]. However, the present experiments were not designed to show the absolute signal level achievable for biotin binding, but rather the sensitivity enhancement achievable using dye labelling.

Even though the binding of even small molecules in many cases can be detected using standard SPR, such monitoring often requires specialized and unfavourable conditions. The detection often requires immobilization of a large amount of binding partner, e.g., protein, on the sensor slide surface and a high density of surface binding sites, which may cause problems with steric effects and crowding (and even avidity effects for multivalent binders) [28]. In the important application field of drug screening on cell membrane receptors, for example, the densely packed environment in a dextran matrix is far from the biologically relevant environment of physically isolated receptor proteins in an essentially flat membrane surface. Also, as is well known, a high density of surface sites regularly causes problems with mass transport limitation effects in kinetic analysis [3], and in particular so since the diffusion is further restricted in a dense matrix as compared to free solution. Standard SPR detection in drug or fragment screening generally requires high concentrations of the screened compounds in solution to enhance the detectability [29,30], which leads to solubility problems and high non-specific binding. Consequently, it would be desirable to enhance the sensitivity of SPR detection of small molecules for a number of reasons.

The high concentration of native biotin as compared to labelled biotin in the present experiments was used to ensure saturation of native biotin binding, and to eliminate the risk that labelled biotin would yield a stronger signal simply due to a higher binding constant to the surface. However, this risk was later ruled out during the experiments presented in Section 4.2.

### 3.2. Small Molecule Assay: Linearity and Competition

**Figure 3.**
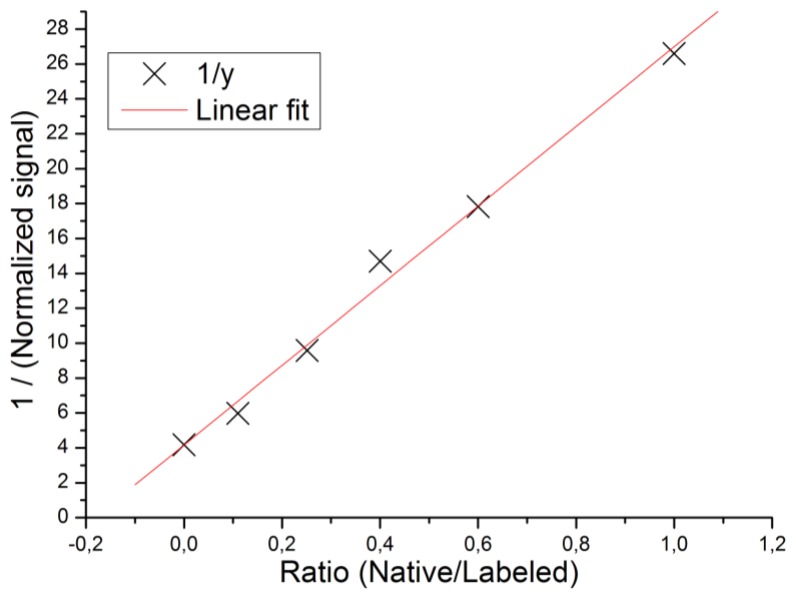
Equilibrium interaction analysis of the competitive binding of native biotin and labelled biotin. The data points are described well by a linear regression line (R^2^ = 0.992) as predicted by Equation (6). The intersection and slope of the linear regression line were used to calculate the relative equilibrium binding constant of labelled biotin.

A second set of experiments was designed to show the linearity of the label-enhanced SPR method and its applicability to competitive analysis. Mixtures of varying ratios of native and labelled biotin, but at a constant total biotin concentration of 50 μM, were injected onto avidin sensor slides. The sensorgrams were registered in the enhanced mode, which only measures the binding of the dye-labelled biotin. The results are presented in Table 1 and in Figure 3, in which the inverted signal is plotted *vs.* the native/labelled biotin ratio. The standard deviation (of the inverted signal) between replicates was 0.38 units in these experiments. The residual experimental variability around the linear regression model in Figure 2 was 0.8%. Since the data in Figure 3, in accordance with the theory of competitive analysis (*cf.* Equation (6)), yields a straight line, the experiments serve to show that the label-enhanced SPR concept is applicable to competitive analysis. Thus, even though the biotin binding cannot be directly detected in the present system according to Section 4.1, the concentration of biotin in solution can be indirectly determined using competitive analysis. The competitive format has the potential to improve the detection limit of small molecules using SPR by about 100×.

Based on the very plausible assumption that the binding of the small biotin molecules to well-defined, discrete binding sites on the avidin molecules follows a competitive Langmuir adsorption isotherm, the data may be fitted to the following equation [31]:

(6) $$1/\text{Signal}=1/\left(\text{KC}\right)+\left(\text{b}/\text{a}\right){\text{k}}_{\text{b}}/\left({\text{k}}_{\text{a}}\text{K C}\right)$$

where C is the total surface binding capacity of biotin, K is an arbitrary sensitivity constant of labelled biotin, k_a_ and k_b_ are the equilibrium adsorption constants of labelled and native biotin, respectively, and a and b are the solution concentrations of labelled and native biotin, respectively. The ratio of the binding constants, k_b_/k_a_, calculated from Equation (6), was 5.5, indicating that the affinity of native biotin is 5.5 times higher than that of the labelled biotin. This difference is not unexpected, since the biotin binding sites of avidin are expected to show the highest binding affinity for native biotin, but, most importantly, this affinity difference does not reduce the practical usefulness of the competitive assay format.

The applicability to different assay formats is of critical importance for the usability of label-enhanced SPR. In many cases, when analysing complex samples of biochemical origin, it may simply not be possible to selectively label only the analyte, and in other cases, when there is a risk that the label will interfere with the binding event, it may not be desirable to label the analyte. In such cases, labelling of an analyte analogue combined with indirect monitoring of the binding event in a competitive assay may be used. As is well established, competitive assays can be used to determine both concentration, affinity constants, and kinetic reaction constants [32–34]. To measure concentration, also inhibitive assays (a.k.a. competition in solution) and sandwich assays may be used. Both of these assay formats use a binder of the analyte which is labelled [6].

The good linear fit of the data in Figure 3 to Equation (6) also serves to justify the linear approximations made in Equations (2) and (4) in Section 2. The higher affinity for the native biotin as compared to the labelled biotin obtained from the competition assay verifies that the risk discussed in Section 4.1, *i.e.*, that the high signal obtained for labelled biotin would be due to a higher affinity for labelled biotin compared to the native biotin, can be safely ruled out.

### 3.3. DNA Hybridization Assay: Specificity

**Figure 4.**
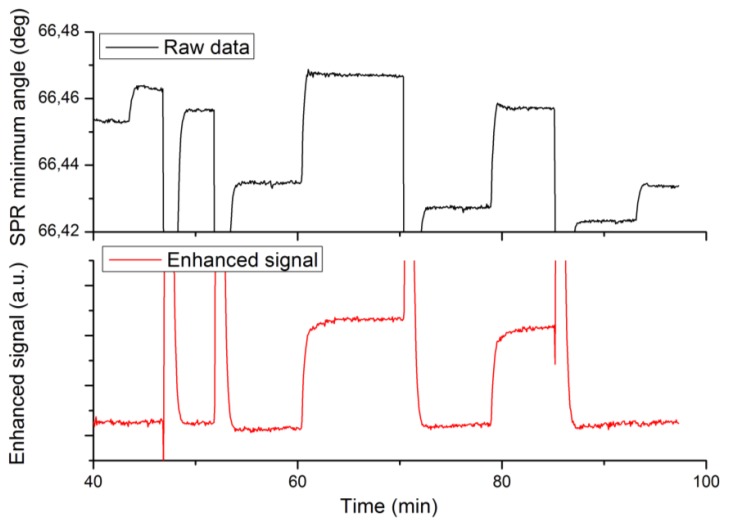
Standard (upper panel) and enhanced (lower panel) sensorgrams of the hybridization of native and labelled DNA oligonucleotides. The enhanced sensorgram has been calculated from the standard SPR sensorgram according to Equation (5). Native DNA is injected at 44 and 93 min. Labelled DNA is injected at 60 and 79 min. Sodium hydroxide regeneration solution was injected at 47, 52, 70, and 85 min.

A third set of experiments was designed to validate the enhanced specificity of label-enhanced SPR. Native and labelled 25-mer DNA oligonucleotides were alternately hybridized to a complementary strand immobilized on the sensor slide surface. The hybridization sensorgrams are shown in Figure 4, where the upper panel shows the standard SPR sensorgram and the lower panel shows the enhanced sensorgram. Note that both sensorgrams represent the same run. The different injections are detailed in the figure caption.

The most obvious feature of Figure 4 is the high specificity of labelled DNA as compared to native DNA in the enhanced sensorgram. There is a strong, stable signal from the labelled DNA (injections 2 and 3 at times 60 and 79 min), but no discernible signal from the native DNA (injections 1 and 4 at times 45 and 93 min). There are remaining features from the sodium hydroxide regeneration injections even in the enhanced sensorgram. This is due to the fact that these injections cause very large disturbances of the refractive index, which obviously fall outside the linear range of the method.

The second feature is the absence of baseline drift in the enhanced sensorgram. This drift, which most probably is due to desorption of loosely bound protein from the surface during sodium hydroxide injections, is clearly seen in the standard sensorgram. The improved stability of the baseline in the enhanced sensorgram is another corollary of the high specificity, since the desorbed protein in itself does not absorb light at the SPR wavelength used.

The signal enhancement effect is less apparent in this case than in the case of biotin in Sections 4.1–4.2. The reason for this is that the oligonucleotides as such are much heavier (about 10 kDa) than the biotin (244 Da), thereby causing a relatively stronger signal in the standard sensorgram. Still, the signal-to-noise ratio of the labelled DNA in the enhanced sensorgram is 2.5 times higher than that of the native DNA in the standard sensorgram. There are many applications where the sensitivity of standard SPR may be adequate, but where the improved specificity offered by dye-labelled SPR may be a pronounced advantage, e.g., when analysing large proteins, like antibodies at low concentration, in samples showing a high non-specific binding of proteins.

### 3.4. DNA Hybridization Assay: Noise Reduction

**Figure 5.**
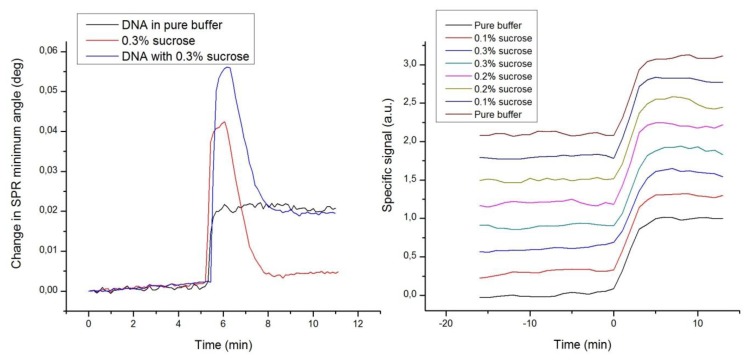
(**Left**): SPR minimum angle sensorgrams of labelled DNA, 0.3% sucrose, and labelled DNA mixed with 0.3% sucrose; (**Right**): Enhanced sensorgrams of labelled DNA mixed with different concentrations of sucrose. All the enhanced sensorgrams overlap, but an offset has been added to the ordinate for clarity.

A fourth set of experiments was designed to demonstrate the immunity of label-enhanced SPR to noise emanating from variations of the bulk solution environment, or more specifically from variations in the bulk liquid composition. Repeated hybridizations of dye-labelled DNA to a complementary DNA strand immobilized on the sensor slide surface were performed with different concentrations of sucrose added to the buffer. The sucrose additions were selected to represent large variations in the composition, and consequently the refractive index, of the buffer. Figure 5 shows standard sensorgrams of DNA only, sucrose only, and DNA with added sucrose, as well as enhanced sensorgrams of a number of DNA hybridizations in the presence of varying amounts of sucrose.

Figure 5 clearly shows that the strong disturbances from the sucrose additions in the standard sensorgrams are absent in the enhanced sensorgrams. The large bulk refractive index variations induced by sucrose are efficiently eliminated, which is yet another embodiment of the high specificity of label-enhanced SPR. It is rather apparent that the hybridization kinetics, obscured in the standard sensorgrams, can easily and robustly be quantified in the enhanced sensorgrams. The DNA hybrids are so stable that the hybridization level can still be measured after the passing of the injection pulse, but for fast associating or dissociating species, it is essential to be able to discern the surface interaction process from the refractive index disturbance caused by the sample injection pulse.

Most often, it is impossible to exactly match the bulk refractive index of the injected sample with that of the running buffer. This problem is especially pertinent in connection with small molecule drug screening or fragment screening using SPR [29,35], where fast and weak interactions combined with the inherent low signal levels of small molecules may cause detection problems. The compounds to be screened are most often stored in DMSO, and it is practically impossible to exactly match the DMSO concentration of all samples with that of the running buffer. Therefore, bulk disturbances are commonly encountered in standard SPR. There are existing procedures for minimizing the influence of bulk disturbances in standard SPR, but these require double or triple referencing with a reference flow channel, blank samples and the creation of calibration curves composed of several injections of DMSO at different concentrations [36,37]. It is quite clear that there is a great interest in simplifying these procedures in order to decrease the complexity, experimental time, and uncertainty of drug screening.

The present experiments demonstrate how refractive index variations due to composition variations of the bulk solution can be efficiently eliminated using label-enhanced SPR. Other dominating noise factors in SPR originate from temperature and pressure variations of the bulk solution. Since such variations cause the same kind of bulk refractive index variations as do composition variations, it seems safe to assume that also temperature and pressure noise can be efficiently reduced, even though this is not explicitly demonstrated by the experiments.

### 3.5. A Comparison of Signal Enhancement Techniques for SPR

There are a number of suggestions in the literature on the use of heavy labels, e.g., metal and plastic nanoparticles or high molecular weight compounds, to enhance assay sensitivity by a mere mass increase [38–40]. However, these suggestions show a number of limitations as compared to dye-label enhancement. Firstly, and most importantly, non-absorbing labels do not offer the improved specificity that absorbing labels do. Secondly, slow diffusive mass transport and steric hindrance exclude the use of heavy labels in kinetic analysis. Thirdly, the steric and entropic interference with biochemical binding events is much more pronounced for large labels, like e.g., latex particles, than for dye labels that are small molecules, excluding the use of heavy labels in equilibrium analysis. And fourthly, simple and reliable methods to achieve a controlled 1:1 coupling of biomolecules to larger particles are scarce, while there exists a rich and well-established range of methods for the controlled labelling and purification of molecular species [41]. Consequently, the use of heavy labels to increase the analytical sensitivity in SPR is generally limited to specialized cases of mere concentration analysis.

Another way to improve the sensitivity of SPR is to utilize surface plasmon fluorescence. However, fluorescence methods are plagued by the quenching problems discussed in Section 1 [3,15], while quantitative label-enhanced SPR, based on absorbance of light rather than on fluorescence, offers a much more robust method that is immune to quenching problems. Also, the highly specific nature of fluorescence detection does not allow the monitoring and quantification of non-labelled binding steps, like e.g., the important receptor immobilization step. It would, in principle, be possible to combine standard SPR and surface plasmon fluorescence in one single instrument [42], but such an instrument would be optically complex and expensive. To the best of the authors' knowledge, no such combined commercial instrument exists today.

The sensitivity enhancement effect demonstrated using label-enhanced SPR is, quite naturally, largest for small analyte molecules. In the present work, we demonstrate a 100× increase of the achievable signal-to-noise ratio for the 244 Da biotin model molecule. For larger analyte molecules, with a molecular weight of a few kDa, e.g., peptides or oligonucleotides, the sensitivity increase is on the order of 10×, which is still a sizeable improvement for critical applications. For full size proteins, larger than about 25 kDa, no signal enhancement effect is obtained using a single dye label, but in this case multiple labelling may be used to further enhance the signal. However, such large molecules usually generate enough signal to be conveniently analysed by conventional SPR. Nevertheless, the improved specificity is a benefit of label-enhanced SPR irrespective of the size of the analyte molecule. This improved specificity may be advantageous also in the analysis of antibodies and other heavy proteins in crude samples, e.g., serum samples or culture media samples, with a high level of non-specific binding.

## 4. Conclusions

The label-enhanced SPR method can improve the analytical sensitivity of small-molecule detection in SPR about 100-fold. By working in the competitive mode, the detection limit in small molecule analysis can be improved analogously even without direct labelling of the analyte. The method exhibits a high specificity with respect to the labelled compound, which may be of importance also in assays where the sensitivity of standard SPR is adequate, but where there is a high level of non-specific binding. A consequence of the high specificity is also that noise emanating from refractive index variations of the bulk liquid is eliminated. Since label-enhanced SPR can be run simultaneously and in parallel with conventional SPR, all the capabilities of label-free SPR and label-enhanced SPR can be combined on one standard SPR instrument platform. Consequently, the combination of label-free and label-enhanced SPR is foreseen to significantly widen the application field of optical biosensors within biotechnology, biochemistry, and drug discovery.

## Figures and Tables

**Table 1.** Results of labelled and native biotin competitive interaction assay. The amount of immobilized avidin was used to normalize the label-enhanced SPR signal. The ratio (b/a) is the ratio of native biotin concentration (b) to labelled biotin concentration (a). The results are plotted in Figure 2.

**b/a**	**Immobilized Avidin** **(mdeg)**	**Enhanced Signal** **(a.u.)**	**Normalized Signal** **(a.u.)**	**1/Normalized Signal** **(a.u. 10^3^)**
0.0	163	39.0	0.2393	4.18
0.1	94	15.7	0.1670	5.99
0.2	138	14.4	0.1043	9.58
0.4	191	13.0	0.0681	14.69
0.6	239	13.4	0.0561	17.84
1.0	117	4.4	0.0376	26.59
